# Differential temporal utility of passively sensed smartphone features for depression and anxiety symptom prediction: a longitudinal cohort study

**DOI:** 10.1038/s44184-023-00041-y

**Published:** 2024-01-04

**Authors:** Caitlin A. Stamatis, Jonah Meyerhoff, Yixuan Meng, Zhi Chong Chris Lin, Young Min Cho, Tony Liu, Chris J. Karr, Tingting Liu, Brenda L. Curtis, Lyle H. Ungar, David C. Mohr

**Affiliations:** 1 Department of Preventive Medicine, Center for Behavioral Intervention Technologies (CBITs), Feinberg School of Medicine, Northwestern University, Chicago, IL USA; 2 Department of Computer and Information Science, University of Pennsylvania, Philadelphia, PA USA; 3 Positive Psychology Center, University of Pennsylvania, Philadelphia, PA USA; 4 Roblox Corporation, San Mateo, CA USA; 5 Audacious Software, Chicago, IL USA; 6 Technology & Translational Research Unit, National Institute on Drug Abuse (NIDA IRP), National Institutes of Health (NIH), Bethesda, MD USA

**Keywords:** Biomarkers, Psychiatric disorders

## Abstract

While studies show links between smartphone data and affective symptoms, we lack clarity on the temporal scale, specificity (e.g., to depression vs. anxiety), and person-specific (vs. group-level) nature of these associations. We conducted a large-scale (*n* = 1013) smartphone-based passive sensing study to identify within- and between-person digital markers of depression and anxiety symptoms over time. Participants (74.6% female; *M* age = 40.9) downloaded the LifeSense app, which facilitated continuous passive data collection (e.g., GPS, app and device use, communication) across 16 weeks. Hierarchical linear regression models tested the within- and between-person associations of 2-week windows of passively sensed data with depression (PHQ-8) or generalized anxiety (GAD-7). We used a shifting window to understand the time scale at which sensed features relate to mental health symptoms, predicting symptoms 2 weeks in the future (distal prediction), 1 week in the future (medial prediction), and 0 weeks in the future (proximal prediction). Spending more time at home relative to one’s average was an early signal of PHQ-8 severity (distal *β* = 0.219, *p* = 0.012) and continued to relate to PHQ-8 at medial (*β* = 0.198, *p* = 0.022) and proximal (*β* = 0.183, *p* = 0.045) windows. In contrast, circadian movement was proximally related to (*β* = −0.131, *p* = 0.035) but did not predict (distal *β* = 0.034, *p* = 0.577; medial *β* = −0.089, *p* = 0.138) PHQ-8. Distinct communication features (i.e., call/text or app-based messaging) related to PHQ-8 and GAD-7. Findings have implications for identifying novel treatment targets, personalizing digital mental health interventions, and enhancing traditional patient-provider interactions. Certain features (e.g., circadian movement) may represent correlates but not true prospective indicators of affective symptoms. Conversely, other features like home duration may be such early signals of intra-individual symptom change, indicating the potential utility of prophylactic intervention (e.g., behavioral activation) in response to person-specific increases in these signals.

## Introduction

Technological advances facilitating personal sensing, or passively collected signals from networked smartphone sensors^1^, stand to address critical gaps in measuring and treating affective symptoms. Features assessed using smartphones could signal novel treatment targets; for example, the daily number of calls and texts made may signal changes in social behavior relevant to depression^2^. Similarly, personal pronoun use in text messages has been linked with depression and anxiety symptoms^3–5^, and reductions in I-pronoun use track broad improvements in therapy^6^. Incorporating sensed data into clinical care may also enhance shared decision-making^7^. For instance, deviations in GPS-location-based features could signal relevant changes to patient depression severity that could trigger a provider notification. Finally, better understanding how personal sensing can be leveraged to reliably signal current or prospective deterioration may address a key question about existing digital mental health interventions^8,9^, which is how best to optimize the delivery of intervention components so that the right component is received at the right time, while minimizing user burden^10–13^.

As a foundational step in realizing this potential, studies have evaluated how sensed features relate to affective symptom severity. Prior work shows that different sensor signals such as the number and type (i.e., incoming or outgoing) of phone calls and text messages relate to affective symptoms^14,15^. Additional data suggest that the content of text messages predicts mood and anxiety symptoms^3–5,16^. Even mobile phone keystroke patterns have been associated with mood states^17^. Other smartphone signals such as GPS-location-derived features have demonstrated associations with affective symptoms across many different studies^4,14,18,19^; however, due to challenges with replication and generalizability, there are calls for these findings to be replicated in larger and more heterogeneous samples^19,20^.

Additional challenges stem from the dearth of studies on how temporal characteristics impact observed relationships between sensed features and symptoms, including the data window (i.e., interval over which sensor data are collapsed) and time lag (i.e., time between of predictor and outcome measurement). Previous studies of mental health outcomes have used 24-h data windows to predict mental health outcomes lagged by short timeframes such as 1 h or 1 day^21^. Other studies have used slightly larger data windows to predict mental health outcomes at lags of 1 or 2 weeks in the future^3,22,23^. The predictive power of different sensor types may be more or less clinically meaningful depending on the data window and time lag used^22^. For example, a recent study we conducted of text message language features as they related to depression symptom severity demonstrated that a data window of 4 weeks was the optimal aggregation for prediction^5^. Another example in social media data indicated that using a data window of 2 months to predict depression severity with time lags of between 2 and 4 weeks was the optimal analytic setup^24^. Understanding how the relationships between sensed features and affective symptoms change depending on data windows and time lags is essential to informing the clinical utility of sensed data for mental health.

Our primary objective for this study was to evaluate smartphone sensor-based markers that prospectively relate to depression and anxiety symptoms. We examined sensed features’ prospective relationships to symptom severity for depression and anxiety, as well as their utility as distal or proximal predictors of affective symptom severity, using a shifting 2-week sensor data window across various time lags to predict future affective symptoms.

## Methods

### Participants

Participants were recruited in 3 waves, with a total of 1,093 enrolled. Participants in wave 1 (July–September 2019) were recruited from the Center for Behavioral Intervention Technologies (CBITs) Health research registry and ResearchMatch.org, a national health volunteer registry supported by the National Institutes of Health. Participants in wave 2 (February–April 2020) were recruited from the CBITs Health and ResearchMatch.org registries, as well as from Focus Pointe Global, a market research data collection company. Participants in wave 3 (January–April 2021) were recruited from digital advertisements (e.g., posts on Instagram, Facebook, Twitter, craigslist, etc.), the CBITs Health and ResearchMatch.org registries, and Focus Pointe Global.

Inclusion and exclusion criteria for waves 1 and 2 did not differ. We conducted stratified sampling based on baseline PHQ-8 scores such that a minimum of 50% experienced at least moderate depression symptoms (PHQ-8 ≥ 10). In Wave 3, all participants were recruited to have at least moderate depression symptoms (PHQ-8 ≥ 10). Across all waves, participants were required to be at least 18 years old, a U.S. resident, able to read English, and own an Android smartphone with an active data and text messaging plan. Participants were excluded if they self-reported a diagnostic history of bipolar disorder, manic, or hypomanic episode, schizophrenia, or other psychotic disorder.

Participants were compensated up to $142 for completion of assessments, as well as bonuses delivered at the end of each assessment week for participants who were running the latest version of the app and had transmitted sensed data within the past 2 days.

### Procedure

After providing written informed consent, participants enrolled in the study for 16 weeks. All participants downloaded the LifeSense app^25^, which automatically collected GPS-based sensor data, app, and device use data, and communication data from participants’ smartphones (see Supplementary Table S1 for a list of sensors used and frequency acquired, consistent with Saeb et al., 2015). Participants responded to web-based surveys (e.g., GAD-7)^26^ through the REDCap platform at baseline and every 3 weeks thereafter (i.e., weeks 1, 4, 7, 10, 13, 16)^27,28^. Participants also completed PHQ-8 surveys via the LifeSense app at the beginning and end of every third week in the study^29^. Because of this cadence, PHQ-8 instructions were modified to ask participants about their symptoms over the past week rather than past two weeks. All procedures were approved by the Northwestern University Institutional Review Board.

### Analytic methods

**Fig. 1:**
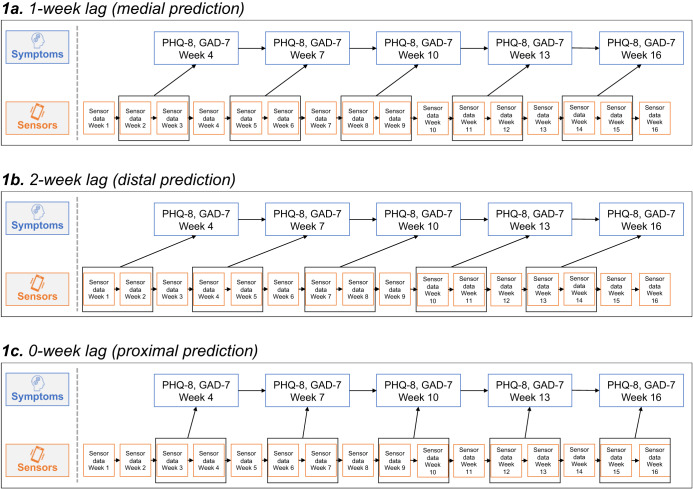
Timing of associations between sensed data and affective symptoms. Testing the influence of past 2 week sensor data on subsequent week depression and anxiety symptoms (**1a**, medial prediction, 1-week lag), as well as the effect of shifting the sensor data time window on symptom prediction (**1b**, distal prediction, 2-week lag; and **1c**, proximal prediction, 0-week lag). The orange boxes in each panel depict the sliding sensor window across various lag times.

Multilevel regression models were tested in R using the *lmerTest* package with maximum likelihood estimation^30^. Specifically, we evaluated the associations of clustered sensor features aggregated over a 2 week window (see Supplementary Table S2 for details on clustering) with subsequent depression and anxiety symptoms. The 2 week window was selected for three reasons: to permit sufficient density of sensor data, to align with gold-standard assessments of depression and anxiety symptoms that ask about the past 2 weeks^26,29^, and to be consistent with prior sensing studies^4,31,32^. The prediction window was shifted such that three different models were tested for each outcome: (1) medial prediction is at a 1-week lag (Fig. 1a), (2) distal prediction is shifted back 1 week for a 2-week lag (Fig. 1b), and (3) proximal prediction is shifted forward 1 week for a 0-week lag (Fig. 1c). While there was no overlap between the sensor window and symptom reporting for distal or medial prediction, proximal prediction involved taking sensor data from the week immediately before and the week concurrent with symptom reporting (e.g., weeks 3 and 4 of sensor data predicting the week 4 symptom assessment). Sensor predictors were person-mean centered, and for each sensor predictor, both a person mean term and a within-person deviation term were included in the model. Additional model terms included time (week; centered around zero), the random intercept, and the demographic covariates of age (centered), gender, and urbanicity/rurality. See Supplementary Materials for more detail on modeling.

## Results

### Data aggregation and demographics

Data were available from 1013 participants (74.6% female; mean age = 40.9 years [*SD* = 12.7]), including a total of 4731 PHQ-8 scores (of 5065 possible; 6.59% missing) and 4649 GAD-7 scores (of 5065 possible; 8.21% missing). Table 1 contains complete demographic data.

**Table 1:** Demographic data.

Variable	All waves (*n* = 1013)	Wave 1 (*n* = 265)	Wave 2 (*n* = 332)	Wave 3 (*n* = 416)
Age in years, mean (sd)	40.91 (12.69)	38.89 (12.01)	43.06 (12.26)	40.48 (13.22)
Sex at birth, *n* (%)				
Female	756 (74.63%)	207 (78.11%)	241 (72.59%)	308 (74.04%)
Male	257 (25.37%)	58 (21.89%)	91 (27.41%)	108 (25.96%)
Gender identity, *n* (%)				
Woman	732 (72.26%)	197 (74.34%)	240 (72.29%)	295 (70.91%)
Man	253 (24.98%)	58 (21.89%)	91 (27.41%)	104 (25.00%)
Non-binary	15 (1.48%)	6 (2.26%)	1 (0.30%)	8 (1.92%)
Transgender	7 (0.69%)	0 (0%)	0 (0%)	7 (1.68%)
Unknown	6 (0.59%)	4 (1.51%)	0 (0%)	2 (0.48%)
Race, *n* (%)				
White	804 (79.37%)	211 (79.62%)	254 (76.51%)	339 (81.49%)
Black/African American	107 (10.56%)	20 (7.55%)	52 (15.66%)	35 (8.41%)
Asian	33 (3.26%)	9 (3.40%)	6 (1.81%)	18 (4.33%)
Native American/Alaska Native	10 (0.99%)	3 (1.13%)	4 (1.20%)	3 (0.72%)
More than one Race	53 (5.23%)	20 (7.55%)	15 (4.52%)	18 (4.33%)
Unknown	6 (0.59%)	2 (0.75%)	1 (0.30%)	3 (0.72%)
Ethnicity, *n* (%)				
Hispanic/Latinx	78 (7.7%)	25 (9.43%)	19 (5.72%)	34 (8.17%)
Non-Hispanic/Non- Latinx	932 (92.0%)	238 (89.81%)	313 (94.28%)	381 (91.59%)
Unknown	3 (0.30%)	2 (0.75%)	0 (0%)	1 (0.24%)
Highest level education completed, *n* (%)				
Some high school, no diploma	14 (1.38%)	3 (1.13%)	4 (1.20%)	7 (1.68%)
High school/GED	77 (7.60%)	12 (4.53%)	28 (8.43%)	37 (8.89%)
Some college, no degree	233 (23.0%)	42 (15.85%)	72 (21.69%)	119 (28.61%)
Associate’s degree	163 (16.09%)	37 (13.96%)	68 (20.48%)	58 (13.94%)
Bachelor’s degree	312 (30.80%)	94 (35.47%)	99 (29.82%)	119 (28.61%)
Master’s Degree	174 (17.18%)	59 (22.26%)	53 (15.96%)	62 (14.90%)
Professional Degree	19 (1.88%)	7 (2.64%)	5 (1.51%)	7 (1.68%)
Doctoral Degree	19 (1.88%)	11 (4.15%)	2 (0.60%)	6 (1.44%)
Unknown	1 (0.10%)	0 (0%)	1 (0.30%)	0 (0%)
Marital status, *n* (%)				
Single/never married	338 (33.37%)	95 (35.85%)	106 (31.93%)	137 (32.93%)
Domestic partnership	158 (15.60%)	45 (16.98%)	51 (15.36%)	62 (14.90%)
Married	335 (33.07%)	88 (33.21%)	107 (32.23%)	140 (33.65%)
Separated	32 (3.16%)	5 (1.89%)	12 (3.61%)	15 (3.61%)
Divorced	143 (14.12%)	29 (10.94%)	55 (16.57%)	59 (14.18%)
Unknown	7 (0.69%)	3 (1.13%)	1 (0.30%)	3 (0.72%)
Household income, *n* (%)				
<$10,000	67 (6.61%)	12 (4.53%)	23 (6.93%)	32 (7.69%)
$10,000–19,999	90 (8.88%)	19 (7.17%)	33 (9.94%)	38 (9.13%)
$20,000–39,999	212 (20.93%)	40 (15.09%)	66 (19.88%)	106 (25.48%)
$40,000–59,999	206 (20.34%)	55 (20.75%)	69 (20.78%)	82 (19.71%)
$60,000–99,999	242 (23.89%)	83 (31.32%)	78 (23.49%)	81 (19.47%)
>$100,000	169 (16.68%)	48 (18.11%)	58 (17.47%)	63 (15.14%)
Unknown	27 (2.67%)	8 (3.02%)	5 (1.51%)	14 (3.37%)
Employment, *n* (%)				
Employed	643 (63.47%)	206 (77.74%)	211 (63.55%)	226 (54.33%)
Unemployed	135 (13.33%)	21 (7.92%)	42 (12.65%)	72 (17.31%)
Disability	104 (10.27%)	15 (5.66%)	33 (9.94%)	56 (13.46%)
Retired	49 (4.84%)	10 (3.77%)	16 (4.82%)	23 (5.53%)
Other	78 (7.70%)	12 (4.53%)	29 (8.73%)	37 (8.89%)
Unknown	4 (0.39%)	1 (0.38%)	1 (0.30%)	2 (0.48%)
Baseline PHQ-8				
Minimal (0–4), *n* (%)	140 (13.82%)	54 (20.38%)	86 (25.9%)	0 (0%)
Mild (5–9), *n* (%)	129 (12.73%)	45 (16.98%)	84 (25.3%)	0 (0%)
Moderate (10–14), *n* (%)	330 (32.58%)	89 (33.58%)	68 (20.48%)	173 (41.59%)
Moderate-Severe (15–19), *n* (%)	289 (28.53%)	57 (21.51%)	56 (16.87%)	176 (42.31%)
Severe (20–24), *n* (%)	125 (12.34%)	20 (7.55%)	38 (11.45%)	67 (16.11%)
Baseline GAD-7				
Minimal (0–4), *n* (%)	216 (21.32%)	74 (27.92%)	106 (31.93%)	36 (8.65%)
Mild (5–9), *n* (%)	267 (26.36%)	85 (32.08%)	83 (25.00%)	99 (23.8%)
Moderate (10–14), *n* (%)	267 (26.36%)	61 (23.02%)	69 (20.78%)	137 (32.93%)
Severe (15–21), *n* (%)	257 (25.37%)	44 (16.6%)	72 (21.69%)	141 (33.89%)

### Primary results

Table 2 (PHQ-8) and 3 (GAD-7) present results for all within-person and between-person effects of sensor data on symptoms over time; for parsimony, only features with at least some significant relationships to outcomes are described below in the text.

**Table 2:** Multilevel model results predicting PHQ-8 from sensing data across shifting prediction windows.

Predictor	Sensing predicting PHQ-8 with 2-week lag (*R*^*2*^ = 0.049)			Sensing predicting PHQ-8 with 1-week lag (*R*^*2*^ = 0.048)			Sensing predicting PHQ-8 with 0-week lag (*R*^*2*^ = 0.053)		
	Estimate	SE	*p*-value	Estimate	SE	*p*-value	Estimate	SE	*p*-value
Home duration—B	0.089	0.206	0.666	0.112	0.203	0.582	0.113	0.201	0.575
**Home duration - W**	**0.219**	**0.087**	0.012*	**0.198**	**0.087**	0.022*	**0.183**	**0.091**	0.045*
Circadian movement - B	−0.323	0.266	0.226	−0.189	0.273	0.490	−0.375	0.266	0.159
**Circadian movement - W**	0.034	0.062	0.577	−0.089	0.060	0.138	**−0.131**	**0.062**	0.035*
Location variability - B	−0.030	0.227	0.893	−0.149	0.230	0.516	−0.043	0.226	0.848
Location variability - W	−0.133	0.130	0.306	−0.129	0.129	0.316	−0.110	0.131	0.400
More frequent venues - B	−0.116	0.232	0.619	−0.092	0.232	0.693	−0.073	0.237	0.758
**More frequent venues - W**	−0.064	0.063	0.308	**−0.185**	**0.062**	0.003**	**−0.168**	**0.063**	0.007**
Less frequent venues - B	−0.345	0.213	0.106	−0.244	0.215	0.256	−0.295	0.220	0.179
Less frequent venues - W	−0.022	0.060	0.714	−0.069	0.058	0.234	−0.019	0.056	0.729
**GPS variability and mobility - B**	−0.464	0.258	0.073	**−0.503**	**0.252**	0.046*	−0.424	0.252	0.093
GPS variability and mobility - W	−0.052	0.050	0.302	−0.086	0.050	0.083	−0.037	0.050	0.458
Call and text communication - B	−0.079	0.206	0.702	−0.181	0.211	0.391	−0.197	0.211	0.350
Call and text communication - W	−0.077	0.081	0.340	−0.043	0.081	0.592	−0.049	0.079	0.534
App-based messaging - B	0.317	0.235	0.178	0.373	0.238	0.117	0.408	0.238	0.087
**App-based messaging - W**	0.059	0.067	0.385	0.115	0.066	0.083	**0.162**	**0.066**	0.015*
Social media - B	−0.289	0.211	0.171	−0.157	0.210	0.454	−0.241	0.206	0.243
Social media - W	−0.008	0.070	0.904	0.022	0.069	0.753	0.019	0.068	0.785
**Screen-on time - B**	**0.503**	**0.208**	0.016*	0.272	0.211	0.196	**0.541**	**0.214**	0.012*
Screen-on time - W	−0.009	0.050	0.857	0.000	0.047	0.995	0.037	0.047	0.424
Browser - B	0.219	0.206	0.287	0.308	0.206	0.135	**0.367**	**0.205**	**0.075**
Browser - W	−0.052	0.065	0.430	−0.032	0.064	0.617	0.024	0.064	0.709
Email - B	−0.107	0.199	0.592	−0.132	0.203	0.517	−0.102	0.200	0.611
Email - W	0.076	0.068	0.269	0.026	0.064	0.689	0.030	0.065	0.647
Game - B	0.006	0.205	0.978	−0.007	0.203	0.974	−0.044	0.203	0.826
Game - W	0.017	0.058	0.775	0.024	0.059	0.681	−0.028	0.059	0.634
**Launcher - B**	**−0.596**	**0.223**	0.008**	**−0.525**	**0.222**	0.018*	**−0.653**	**0.224**	0.004**
**Launcher - W**	−0.040	0.069	0.561	−0.111	0.070	0.115	**−0.161**	**0.071**	0.023*
**Age**	**−0.603**	**0.174**	0.001**	**−0.573**	**0.174**	0.001**	**−0.581**	**0.173**	0.001**
**Male (vs0. female)**	**−0.521**	**0.169**	0.002**	**−0.542**	**0.170**	0.001**	**−0.563**	**0.169**	0.001**
**Urban (vs0. rural)**	−0.311	0.168	0.065	−0.320	0.168	0.058	−0.307	0.168	0.068
**Study week**	**−0.119**	**0.026**	<0.001***	**−0.107**	**0.026**	<0.001***	**−0.112**	**0.025**	<0.001***
(Intercept)	9.458	0.163	0.000	9.467	0.163	0.000	9.460	0.163	0.000

Features highlighted in bold have at least one significant relationship to the outcome.

B between, W within.

*p < 0.05, **p < 0.01, ***p < 0.001.

#### Location features

Spending more time at home relative to one’s own average (i.e., within-person) was associated with increased future PHQ-8 severity across prediction windows (distal *β* = 0.219, *p* = 0.012; medial *β* = 0.198, *p* = 0.022; proximal *β* = 0.183, *p* = 0.045). Within-person time spent at home was not significantly associated with GAD-7 severity across any of the time windows (Table 2). We observed no evidence that between-person effects for time spent at home were related to PHQ-8 or GAD-7 severity. People with greater GPS variability and mobility less severe next-week PHQ-8 (medial *β* = −0.503, *p* = 0.046), but this signal was absent for distal (*β* = −0.464, *p* = 0.073) and proximal (*β* = −0.424, *p* = 0.093) associations. Table 3.

**Table 3:** Multilevel model results predicting GAD-7 from sensing data across shifting prediction windows.

Predictor	Sensing predicting GAD-7 with 2-week lag (*R*^*2*^ = 0.058)			Sensing predicting GAD-7 with 1-week lag (*R*^*2*^ = 0.056)			Sensing predicting GAD-7 with 0-week lag (*R*^*2*^ = 0.057)		
	Estimate	SE	*p*-value	Estimate	SE	*p*-value	Estimate	SE	*p*-value
Home duration - B	−0.154	0.209	0.460	−0.089	0.206	0.665	−0.115	0.204	0.575
Home duration - W	−0.009	0.107	0.932	−0.066	0.106	0.537	−0.126	0.112	0.261
Circadian movement - B	−0.335	0.275	0.223	−0.142	0.281	0.614	−0.331	0.273	0.226
Circadian movement - W	0.015	0.076	0.847	0.014	0.074	0.847	−0.021	0.077	0.783
Location variability - B	0.111	0.232	0.633	−0.026	0.235	0.913	0.064	0.231	0.783
Location variability - W	−0.263	0.159	0.099	−0.165	0.158	0.297	−0.146	0.161	0.366
More frequent venues - B	−0.277	0.238	0.245	−0.182	0.233	0.435	−0.169	0.236	0.472
More frequent venues - W	0.129	0.077	0.092	−0.052	0.076	0.493	−0.073	0.078	0.352
Less frequent venues - B	−0.151	0.216	0.485	−0.077	0.218	0.722	−0.106	0.224	0.635
Less frequent venues - W	0.109	0.074	0.138	0.010	0.072	0.887	−0.006	0.069	0.935
GPS variability and mobility - B	−0.109	0.262	0.679	−0.235	0.256	0.358	−0.128	0.257	0.617
GPS variability and mobility - W	−0.107	0.061	0.080	−0.089	0.061	0.149	0.061	0.062	0.324
Call and text communication - B	−0.028	0.209	0.894	−0.074	0.214	0.729	−0.076	0.215	0.725
**Call and text communication - W**	**0.279**	**0.099**	0.005**	**0.386**	**0.100**	<0.001***	**0.293**	**0.098**	0.003**
**App-based messaging - B**	**0.486**	**0.237**	0.041*	**0.481**	**0.240**	0.046*	0.466	0.240	0.053
App-based messaging - W	0.137	0.083	0.097	0.121	0.082	0.142	0.067	0.082	0.414
Social media - B	−0.237	0.212	0.264	−0.178	0.212	0.402	−0.257	0.211	0.222
Social media - W	0.012	0.088	0.887	0.082	0.085	0.339	0.066	0.085	0.437
Screen-on time - B	0.282	0.184	0.125	0.247	0.241	0.305	0.272	0.246	0.268
Screen-on time - W	−0.051	0.063	0.422	−0.041	0.056	0.469	0.013	0.057	0.823
Browser - B	0.077	0.208	0.710	0.132	0.208	0.526	0.217	0.209	0.299
Browser - W	0.005	0.080	0.953	−0.035	0.078	0.653	0.004	0.078	0.963
Email - B	0.023	0.202	0.909	−0.008	0.206	0.968	0.010	0.204	0.960
Email - W	0.084	0.084	0.316	−0.004	0.080	0.963	0.019	0.081	0.818
Game - B	0.013	0.206	0.949	−0.016	0.205	0.938	−0.010	0.205	0.961
Game - W	0.077	0.072	0.284	0.046	0.073	0.528	0.017	0.073	0.814
Launcher - B	−0.219	0.226	0.333	−0.216	0.230	0.348	−0.260	0.233	0.264
Launcher - W	−0.121	0.085	0.153	−0.130	0.086	0.131	−0.117	0.087	0.180
**Age**	**−1.163**	**0.177**	<0.001***	**−1.135**	**0.177**	<0.001***	**−1.148**	**0.176**	<0.001***
**Male (vs. female)**	**−0.360**	**0.172**	0.036*	**−0.378**	**0.173**	0.029*	**−0.388**	**0.172**	0.024*
**Urban (vs. rural)**	**−0.520**	**0.170**	0.002**	**−0.532**	**0.170**	0.002**	**−0.530**	**0.170**	0.002**
**Study week**	**−0.183**	**0.032**	<0.001***	**−0.171**	**0.032**	<0.001***	**−0.167**	**0.031**	<0.001***
*(Intercept)*	8.807	0.166	0.000	8.811	0.166	0.000	8.813	0.166	0.000

Features highlighted in bold have at least one significant relationship to the outcome.

*B* between, *W* within.

*p < 0.05, **p < 0.01, ***p < 0.001.

Two other sensed location features were reflective of near- or medial-term PHQ-8 severity but did not predict PHQ-8 severity far in the future. First, people spending time in more frequently visited venues relative to their own average were likely to have lower impending or concurrent PHQ-8 scores (medial *β* = −0.185, *p* = 0.003; proximal *β* = −0.168, *p* = 0.007); however, going to more frequently visited venues did not prospectively predict PHQ-8 severity in the more distant future (distal *β* = −0.064, *p* = 0.308). Second, people who showed more circadian movement (i.e., regularity in 24-h movement patterns) relative to their own average just before and at the time of reporting depression symptoms had less severe PHQ-8 scores than those who showed less circadian movement (proximal *β* = −0.131, *p* = 0.035); however, circadian movement did not prospectively predict PHQ-8 severity (distal *β* = 0.034, *p* = 0.577; medial *β* = −0.089, *p* = 0.138).

#### Communication features

People spending more time on messaging apps relative to their own average reported more severe impending or concurrent PHQ-8 symptoms (proximal *β* = 0.162, *p* = 0.015), but this effect was non-significant for distal (*β* = 0.059, *p* = 0.385) and medial (*β* = 0.115, *p* = 0.083) prediction. While we did not see a significant association between within-person app-based messaging and GAD-7 at any of the time points, people engaging in more app-based messaging at the between-person level were more likely to report higher distal (*β* = 0.486, *p* = 0.041) and medial (*β* = 0.481, *p* = 0.046) GAD-7 severity; however, the association of between-person app-based messaging and GAD-7 severity was non-significant for proximal prediction (*β* = 0.466, *p* = 0.053). Additionally, calling and texting more relative to one’s own average was associated with GAD-7 severity across all prediction windows (distal *β* = 0.279, *p* = 0.005; medial *β* = 0.386, *p* < 0.001; proximal *β* = 0.293, *p* = 0.003). There were no significant associations between PHQ-8 and call/text-based communication at either the within-person or between-person level.

#### Other phone use features

People who used the launcher more on average had lower PHQ-8 scores across time windows (distal *β* = −0.596, *p* = 0.008; medial *β* = −0.525, *p* = 0.018; proximal *β* = −0.653, *p* = 0.004). When people used the launcher more relative to their own average, they reported lower impending or concurrent PHQ-8 scores (proximal *β* = −0.161, *p* = 0.023). Launcher use was not found to be associated with GAD-7 severity at the within or between person level. People who on average had more screen-on time tended to have greater distal (*β* = 0.503, *p* = 0.016) and proximal (*β* = 0.541, *p* = 0.012) PHQ-8 severity; however, this association was non-significant for next-week prediction (medial *β* = 0.272, *p* = 0.196).

#### Demographic effects

Higher PHQ-8 and GAD-7 severity were found for younger people (*β*: [0.573–1.163], *p*: [<0.001–0.001]) and women (*β*: [0.360–0.563], *p*: [0.001–0.036]). People living in rural areas reported higher GAD-7 (*β*: [0.520–0.532], *p*: [0.002–0.002]), but not PHQ-8 (*β*: [0.307–0.320], *p*: [0.058–0.068]).

#### Time effects

There was a significant fixed effect of time, such that people reported decreasing PHQ-8 and GAD-7 severity over the course of the study (*β*: [−0.107 to −0.183], *p*: [<0.001 to <0.001]).

#### Overall variability explained

The models explained a modest amount of overall variability in PHQ-8 (distal *R*^*2*^ = 0.049; medial *R*^*2*^ = 0.048; proximal *R*^*2*^ = 0.053) and GAD-7 (distal *R*^*2*^ = 0.058; medial *R*^*2*^ = 0.056; proximal *R*^*2*^ = 0.057) symptom severity.

## Discussion

In the present study, we aimed to identify passively sensed digital markers that relate to future depression and anxiety symptoms at both the within-person and between-person levels, and across multiple time windows. Location features were more strongly linked with depression symptoms, whereas communication features related to both depression and anxiety. Results highlighted the importance of the prediction lag in understanding personally sensed signals of affective symptoms: certain features (e.g., time spent at home) were consistent predictors of symptom severity across more distal and more proximal prediction windows, whereas others (e.g., circadian movement) were only associated with next-week or current symptoms.

Overall, location features—and time spent at home in particular—were more strongly linked with depression symptoms than anxiety symptoms. The most robust predictor of depression symptoms was spending more time at home relative to one’s own average, which signaled that a participant was likely to report increases in depressive symptoms 1–3 weeks later. This aligns with meta-analytic evidence indicating that greater time spent at home is one of the sensed features that most consistently relates to depression^14^. Broadly, spending more time at home may be reflective of reductions in motivation or hedonic capacity^33^; if this is the case, the finding that increases in time spent at home relate to future depression symptoms would align with the notion of anhedonia as an endophenotype of depression^34^.

In contrast to location features, communication features related to both depression and anxiety symptoms, with a dissociation for communication type: messaging apps signaled impending depression, and both messaging apps and calling/texting signaled future anxiety. Social media messaging apps are feature-rich^35^, such that their usage may reflect a range of different behaviors related to depression (e.g., “doomscrolling”; engaging in social comparison; ruminating; checking to see why others didn’t respond to a message), and they tend to involve indirect conversations about a shared visual stimulus. Conversely, calling and texting are feature-poor and primarily facilitate direct communication with others^35^; in the context of anxiety, within-person increases in these forms of communication may signal greater activation or reassurance seeking. In general, there were more consistent associations of communication data with anxiety symptoms than depression symptoms across prediction windows and communication modalities, suggesting that changes in communication—like changes in home duration for depression—may be an especially useful signal for understanding anxiety. While studies have linked changes in calling and texting with depression symptoms in bipolar disorder^36,37^, the absence of an association with depression in our study aligns with prior research reporting null findings around communication changes in unipolar depression^31,38^. Continued replication of these null findings may suggest that changes in call and text based communication are not a useful proxy for the social withdrawal and decreased motivational processes that characterize depression symptoms^39^.

By using multilevel models to disaggregate within- and between-person effects over time, we identified differential relationships of sensed features with affective symptoms across time windows that have implications for identifying novel treatment targets, personalizing digital mental health interventions, and enhancing traditional patient-provider interactions^12^. One of the predominant hypothesized methods for bringing personalized digital mental health interventions to fruition is understanding how personal sensing can be leveraged to reliably signal current or prospective worsening symptoms^8,9^. Our findings underscore that the sensing context and timing (i.e., prediction lag) are critical factors impacting the utility of sensed features as a marker of affective symptoms. For example, prior studies have shown a broad correlation between circadian movement and depression symptoms^31,32^. Given that within-person changes in circadian movement occur immediately before and contemporaneously with depression rather than predicting symptoms further in the future, interventions in response to decreased circadian movement may benefit from strategies focused on more immediate or impending depression symptoms. Conversely, in light of the prospective, within-person relationships between time at home and depression severity, developers may consider deploying prophylactic depression-focused content (e.g., behavioral activation) in response to person-specific increases in these signals. Finally, features that are significantly related to symptoms primarily at the between-person level (e.g., launcher use with PHQ-8 or app-based messaging with GAD-7) are unlikely to be helpful signals for individualized intervention or as signals of deterioration.

It is important to consider these implications in the context of the low overall amount of variance explained (approximately 5–6% across the different outcomes and lags), as compared to the larger effect sizes seen in early sensing studies, generally in small samples^4,31,32^. While we opted to use multilevel models for explainability, future studies may consider machine learning models to optimize variance explained in light of the high dimensionality of sensor data^40,41^; these models may also provide greater insight into prediction accuracy metrics (e.g., rates of false positives and false negatives) to inform algorithms designed to prospectively predict clinical symptoms. Additionally, although we lagged sensors and symptom assessments, these data are still correlational and should not be interpreted as implying causality. To the best of our knowledge, there has been no research to date that has attempted to change these sensed constructs through targeted interventions, which would provide stronger evidence of potential causality. It will also be important for future studies to vary the sensor data window—which we kept consistent at 2 weeks—along with the lag to determine impacts on predictive power, and to better understand the impact of missing data over time on observed relationships. Further, the declaration of a national emergency due to COVID-19 in March 2020 occurred partway through our second wave of data collection. We did not see differences across waves substantial enough to warrant separate analysis by wave. However, the variability in the environment since the onset of COVID-19 may have tempered some of the associations between certain features (e.g., geographic location) and symptoms due to changing routines. Additional limitations are the differences in delivery mechanism and timeframe of reporting instructions for the GAD-7 (REDCap; past 2 weeks) and PHQ-8 (in-app; past week), which may have influenced responses. Finally, given the relative lack of demographic diversity in our sample, it will be important for future studies to test whether these findings generalize across more diverse populations.

Overall, findings from this large-scale mobile sensing study point to location features as important in predicting depression symptoms, and communication features in predicting both depression and anxiety symptoms. The multilevel, longitudinal approach allowed us to identify that features such as home duration were true prospective markers of intraindividual change in depression symptoms, whereas others, such as circadian movement, may be more indicative of impending or concurrent depression symptoms.

### Supplementary Information

Supplementary Materials

## Data Availability

De-identified self-report data (PHQ-8 and GAD-7) will be made available through the NIMH Data Archive at the conclusion of the study. Passively collected data are not publicly available due to potentially identifying information that could compromise participant privacy.

## Code availability

Code for all modeling is available from the authorship team upon request.
