# Supplementary material for: Differential temporal utility of passively sensed smartphone features for depression and anxiety symptom prediction: a longitudinal cohort study
Source: Npj Ment Health Res. 2024 Jan 4;3:1. doi: 10.1038/s44184-023-00041-y (PMC10955925; doi:10.1038/s44184-023-00041-y)
Supplement: Supplementary file 1 — Supplementary Materials [file 44184_2023_41_MOESM1_ESM.docx]

**Supplementary Materials**

**Supplementary Methods**

**Raw sensor data acquisition**

Table S1 contains a list of the feature types, raw sensor inputs, and the range/frequency with which they were acquired. There were generally low rates of missing data across the sensors: Application Launch, 8.5% missing; Calls (Communication), 4.9% missing; SMS (Communication), 6.8% missing; GPS Location, 4.3% missing. Given this, we filled impacted features with the average value of all participants across the past 14 days, such that we did not drop any participant from analyses for missing passive data. Benefits of mean imputation are that the mean is easy to compute and understand; mean imputation is conservative in that can attenuate correlations involving the imputed variable, protecting against false positives but potentially allowing for false negatives.^1^

**Clustering**

Sensor data preprocessing was conducted using Python. We applied clustering to the processed features to reduce the dimensionality of the data. We used the DBSCAN algorithm using Manhattan distance. The three waves of participants were recruited across several years and differ on certain demographic features, potentially introducing systematic batch effects that could make people in different waves not directly comparable. To account for differences across waves, we applied a batch effect correction to the normalized features before clustering.^2,3^ The resulting clusters (Table S2) were adjusted empirically according to the distance between clusters. We aggregated features within each cluster by taking the average.

**Multilevel modeling**

Sample R code for a hierarchical linear model predicting PHQ-8 scores from sensor data is as follows:

PHQ-8 Score ~

More_frequent_venues.between + More_frequent_venues.within + Screen_on_Time.between + Screen_on_Time.within + Communication.between + Communication.within + GPS_Variability_and_Mobility.between + GPS_Variability_and_Mobility.within + Home_duration.between + Home_duration.within + Less_frequent_venues.between + Less_frequent_venues.within + Location_variability.between + Location_variability.within + Circadian_Movement.between + Circadian_Movement.within + Launcher.between + Launcher.within + Social_Media.between + Social_Media.within + Message.between + Message.within + Browser.between + Browser.within + Email.between + Email.within + Game.between + Game.within + Urban_rural_area.between + age.between + gender_male.between + study_week + (1|pid)

Here, "pid" refers to the ID representing different individuals and serves as the grouping variable. The random intercept term "(1 | pid)" indicates that there is a random effect on the intercept of the model for each unique "pid" value. It estimates individual-specific variation in the outcome variable (PHQ-8 or GAD-7) that is not explained by fixed effects such as "Home_duration" or "Location_variability". The random intercept reflects that data points within the same individual (pid) may be more similar to each other than to data points for other individuals, accounting for the correlation or dependency within person.

We applied person-mean centering, which involved centering the level 1 variables within clusters (persons) in order to calculate the within-person deviation term for each sensed feature (i.e., within-person effects). The within-person deviation term refers to the difference between an individual's score at a given time point and their own mean score on that variable, representing the extent to which an individual's response deviates from their typical or average response.

**References**

1. Donders ART, van der Heijden GJMG, Stijnen T, Moons KGM. Review: a gentle introduction to imputation of missing values. *J Clin Epidemiol*. 2006;59(10):1087-1091. doi:10.1016/j.jclinepi.2006.01.014

2. Johnson WE, Li C, Rabinovic A. Adjusting batch effects in microarray expression data using empirical Bayes methods. *Biostatistics*. 2007;8(1):118-127.

3. Behdenna A, Haziza J, Azencott CA, Nordor A. pyComBat, a Python tool for batch effects correction in high-throughput molecular data using empirical Bayes methods. *BioRxiv*. Published online 2020:2020-03.

**Table S1.** List of feature types, raw sensor inputs, and range of acquisition.

| **Feature type** | **Raw sensor input** | **Range** |
| --- | --- | --- |
| Application Launch | App Category | {Books & Reference, Business, Comics, Communications, Education, Entertainment, Finance, Health & Fitness, Libraries & Demo, Lifestyle, Media & Video, Medical, Music & Audio, News & Magazines, Personalization, Photography, Productivity, Shopping, Social, Sports, Tools, Transportation, Travel & Local, Weather} |
|  | Android Package Kit (APK) Name | com.sec.android.app.launcher |
| Communication | Contact Name | - |
|  | Contact Number | - |
|  | Communication Type | {CALL, SMS} |
|  | Communication Direction | {OUTGOING, INCOMING, MISSED} |
| GPS Location | Latitude | [-90, 90] |
|  | Longitude | [-180, 180] |
|  | Altitude | - |
|  | Measurement Accuracy | >= 0 |
| Running Apps | Android Package Kit (APK) Name | e.g., com.google.android.apps.maps; com.facebook.katana |
|  | App Package Category | {Books & Reference, Business, Comics, Communications, Education, Entertainment, Finance, Health & Fitness, Libraries & Demo, Lifestyle, Media & Video, Medical, Music & Audio, News & Magazines, Personalization, Photography, Productivity, Shopping, Social, Sports, Tools, Transportation, Travel & Local, Weather} |
| Screen State | Screen State Change | {True (from OFF to ON), False (from ON to OFF)} |

*Note.* GPS data and running app data were sampled with a frequency of once every 5 minutes. Communication and application launch data involved event-based sampling.

**Table S2.** Clustering of sensor features

| **Domain** | **Cluster name** | **Features** |
| --- | --- | --- |
| Location | Home duration | Total time spent at home |
|  | Circadian movement | Circadian movement (regularity in 24-hour rhythm) |
|  | Location variability | Entropy; number of location clusters; location variance |
|  | Time spent in frequently visited locations | Duration of time spent in frequently visited semantic locations (food, work, exercise, another person's home, shopping, other); usage of maps and fitness apps |
|  | Time spent in infrequently visited locations | Duration of time spent in less frequently visited semantic locations (places of worship, travel, errand, health, education, entertainment) |
|  | GPS variability and mobility | Geographic displacement; velocity; time spent in points of interest (PoIs); transitions to PoIs |
| Communication | Call- and text-based communication | Number of phone calls; number of text messages; duration of phone calls; length of text messages |
|  | App-based messaging | Duration of running messaging apps in the foreground |
|  | Social media | Duration of running social media apps in the foreground |
| Phone use | Screen-on time | Amount of time phone screen is turned on |
|  | Browser | Duration of running browser in the foreground |
|  | Email | Duration of running email apps in the foreground |
|  | Game | Duration of running game apps in the foreground |
|  | Launcher | Duration of running launcher in the foreground |

*Note.* The launcher is used to organize the home screen and app icons on Android phones.
